# Cinnamon Counteracts the Negative Effects of a High Fat/High Fructose Diet on Behavior, Brain Insulin Signaling and Alzheimer-Associated Changes

**DOI:** 10.1371/journal.pone.0083243

**Published:** 2013-12-13

**Authors:** Richard A. Anderson, Bolin Qin, Frederic Canini, Laurent Poulet, Anne Marie Roussel

**Affiliations:** 1 Diet, Genomics, and Immunology Laboratory, Beltsville Human Nutrition Research Center, Agricultural Research Service, United States Department of Agriculture, Beltsville, Maryland, United States of America; 2 Integrity Nutraceuticals International, Spring Hill, Tennessee, United States of America; 3 Army Institute for Research in Biology, Grenoble, France; 4 National Institute for Health, Joseph Fourier University, Grenoble, France; 5 Ecole du Val de Grâce, 1 place Laveran, Paris, France; Virginia Tech, United States of America

## Abstract

Insulin resistance leads to memory impairment. Cinnamon (CN) improves peripheral insulin resistance but its effects in the brain are not known. Changes in behavior, insulin signaling and Alzheimer-associated mRNA expression in the brain were measured in male Wistar rats fed a high fat/high fructose (HF/HFr) diet to induce insulin resistance, with or without CN, for 12 weeks. There was a decrease in insulin sensitivity associated with the HF/HFr diet that was reversed by CN. The CN fed rats were more active in a Y maze test than rats fed the control and HF/HFr diets. The HF/HFr diet fed rats showed greater anxiety in an elevated plus maze test that was lessened by feeding CN. The HF/HFr diet also led to a down regulation of the mRNA coding for GLUT1 and GLUT3 that was reversed by CN in the hippocampus and cortex. There were increases in *Insr*, *Irs1* and *Irs2* mRNA in the hippocampus and cortex due to the HF/HFr diet that were not reversed by CN. Increased peripheral insulin sensitivity was also associated with increased glycogen synthase in both hippocampus and cortex in the control and HF/HFr diet animals fed CN. The HF/HFr diet induced increases in mRNA associated with Alzheimers including *PTEN*, *Tau* and amyloid precursor protein (App) were also alleviated by CN. In conclusion, these data suggest that the negative effects of a HF/HFr diet on behavior, brain insulin signaling and Alzheimer-associated changes were alleviated by CN suggesting that neuroprotective effects of CN are associated with improved whole body insulin sensitivity and related changes in the brain.

## Introduction

The epidemic of insulin resistance associated with obesity, metabolic syndrome, type 2 diabetes (T2DM), and cardiovascular diseases (CVD) is sweeping both developed and emerging countries. It is estimated that 20-30% of the adult population in most countries has the metabolic syndrome with some segments of the population even higher [1]. While insulin resistance is a key feature of the metabolic syndrome, it is likely that a similar number of people as those with the metabolic syndrome also have insulin resistance but do not have three of the four features of the metabolic syndrome including central obesity, dyslipidemia, hypertension and elevated blood sugar. The presence of the metabolic syndrome further raises the risk for developing type 2 diabetes by about 5-fold and persons with the metabolic syndrome are at roughly twice the risk for developing cardiovascular diseases compared with those without the syndrome [1]. Diabetes and cardiovascular complications of insulin resistance are only part of the problem since the insulin resistance syndrome also has consequences on brain function. Increased cognitive alterations are observed in subjects with T2DM and insulin resistance [2-4]. 

There is a large body of evidence linking impaired insulin function and glucose metabolism to the risk of developing Alzheimer’s Disease (AD)-type neurodegeneration [5-8]. Insulin resistance has been implicated in the pathogenesis of AD and the term “type 3 diabetes” has been used to describe AD [9]. Experimental brain diabetes produced by intracerebral administration of streptozotocin shares many features with AD, including cognitive impairment and disturbances in acetylcholine homeostasis. Experimental brain diabetes is also treatable with insulin sensitizer agents that are used to treat T2DM [9]. AD represents a form of diabetes that selectively involves the brain and has molecular and biochemical features that overlap with both type 1 and T2DM and helps to explain the term type 3 diabetes [9]. 

Presently there are no known treatments that can stop AD, present treatments can only slow the progression of the disease. Due to the link of insulin resistance and AD, factors that slow the progression of insulin resistance should have consequences on AD incidence and rate of progression. 

Accumulating studies demonstrating that insulin and insulin signaling mechanisms are important for neuronal survival [5,7,9] and studies demonstrating reduced expression of the insulin receptor and related members of the insulin signaling pathway in patients and animals suffering from impaired brain function and Alzheimer’s disease (AD) illustrate the urgent need for further studies involving insulin resistance and the brain. 

Diets high in fat and (or) fructose contribute prominently to insulin resistance and impaired cognition. Simple sugars and saturated fats are major components of the diet that promote obesity and insulin resistance. The effects of such diets on the brain are poorly understood but insulin resistance may play an important role by acting on brain energy metabolism and neuroprotective mechanisms. Addition of fructose to the water of rats fed a high-fat, high-glucose diet led to insulin resistance and impaired hippocampal synaptic plasticity and cognition in middle-aged rats [10]. We have shown in *in vitro*, animal, and human studies that CN, aqueous extracts of CN, and CN polyphenols not only improve insulin function but also act as antioxidant and anti-inflammatory compounds to counteract the negative effects of insulin resistance and obesity [11-19]. We have also shown that rats fed a high fat/high fructose diet develop insulin resistance that is prevented by addition of CN to the diet [20].

In this study, we postulated that since CN improves peripheral insulin sensitivity in animals fed a high fat/high fructose diet [20] and CN also improves insulin signaling in the liver and muscle associated with glycogen synthesis [21], that brain insulin signaling and behavior should also improve. Studies in animal models of impaired insulin sensitivity are needed to further understand the interactions among insulin, neurobiological processes, short-term memory, and cognition. 

## Materials and Methods

### Cinnamon powder

The cinnamon (CN) powder (*Cinnamomum burmannii*) was purchased from McCormick Spice Co., Baltimore, MD. A water extract of the CN contained more than 5% type A polyphenols with a tetramer, cassiatannin A, with a molecular weight of 1152 and two identified trimers, cinnamtannin B-1 and cinnamtannin D-1, with a molecular weight of 864 plus two unidentified type A trimers with a molecular weight of 864 [17,22,23]. The insulin-related bioactivity of the type A polyphenols has been documented [12,16,17,22,24]. The HPLC separation and identification of the selected peaks of the aqueous cinnamon extract has been reported [25].

### Animals and diets

Ethics Statement : All experimental procedures were reviewed and approved by the Institutional Ethic Committee for Animal Care, Research Center of the French Health Service ( IRBA^-^CRSSA Army Research Center for Health, Grenoble, France) where the animals were housed, (Protocol 2008/02.1). The rats were maintained and handled in accord with the Guide for the Care and Use of Laboratory Rats (NIH, 1985). 

Wistar rats (Charles River, L'Arbresle, France), 5 weeks old, were kept in a temperature controlled room (ambient temperature set at 22±1°C and relative humidity at 40-60%) with a 12 h light/12 h dark cycle (light at 8h00). The rats were housed in individual cages (26 x 40 x 15 cm) to monitor the water and diet intakes. 

The diets were purchased from SAFE (89290, Augis, France). The control diet contained 5% cellulose, 20% casein, 25% corn starch, 25% potato starch, 16% maltodextrin, 4% soybean oil, 3.5% AIN mineral mix, 1% AIN vitamin mix, 0.3% dl methionine and 0.2% choline bitartrate. The HF/HFr diet was similar except the corn starch, potato starch and maltodextrin were replaced by 46% fructose and 20% lard. The rats were adapted and fed Purina chow (SAFE, Augis, France) for three weeks. They were, then, randomly divided into 4 groups and fed *ad libitum* for 12 weeks one of the following four diets: Purina chow as the control diet (C), the high fat/high fructose diet (HF/HFr) (described above) to induce insulin resistance, or the respective diets containing 20 g cinnamon/kg of diet (C+CN or HF/HFr+CN). There were no differences in food intake among the diets. The amount of CN used was based upon our previous study showing a definite effect of 20 g of CN/kg of diet in spontaneously hypertensive rats [26]. One gram, 3 and 6 grams of CN per day were all shown to have beneficial effects in people with type 2 diabetes [11]. 

All measurements were performed after 12 weeks of consumption of the diets. In a preliminary investigation, the effects of the diets on insulin resistance were determined in 10 animals of each group using the hyperinsulinemic euglycemic clamp technique [20]. In this investigation based on the same model, the behavioral tests were carried out in 20 animals of each group. The mRNA expression in the hippocampus and cortex (see below) were evaluated in 8 to 10 rats of the 20 rats belonging to each group.

### Y-maze

The Y-maze test evaluates the ability of an animal to recognize places already explored and its propensity to explore a new place [27]. The Y-maze apparatus was made of gray plastic according Dellu’s specifications [27]: each arm being 50 cm long, 16 cm wide and surrounded by a 32 cm high wall. The apparatus was placed on a floor generating infrared illumination allowing recording under dim light (25 Lux) to reduce anxiety. The Y-maze tests were carried out during the 12^th^ week of diet in a dedicated room. It consisted of two 10-min sessions separated by a 2-hour time lapse. In the first session, the rats could explore freely only 2 arms: the arm where they were put (Entry arm) and one (Known arm) of the 2 other arms placed at the left and right of the Entry arm. During the second session, they could explore the entire device, and the new available arm (Unknown arm). Both sessions were done using the same context: same procedure and same environment with spatial cues placed on the walls. The rats were tested in the order of their randomization rank. However, each group was equally represented in the tests made in the morning (8h-12h) or the afternoon (13h-17h) or according to the initial condition, namely the left or right arm occlusion during the first session.

For testing, the rats were transferred from the home room to the testing room in their own cage without being handled. They were gently placed at the end of the entry arm of the Y-maze, the nose being directed to the wall. The experimenter left the rat alone in the room and launched immediately the video recording using a remote control device. At the end of the session, the number of defecation bolus was counted. Meticulous clean ups were then completed, one with soapy water and one with water/isopropyl alcohol (50%) solution. The behavior was recorded on a computer, locomotion being automatically analyzed using a video tracking device (Videotrack, Viewpoint, Lyon) and rearings being scored by the experimenter (Labwatcher, Viewpoint, Lyon). The global behavior in the test was assessed using (i) the defecation bolus number, (ii) the distance traveled in the whole Y-maze, (iii) the time spent in rearing and, (iv) the percentage of spontaneous choices of the unknown arm in the second session (alternation). The cognitive aspect of the behavior was analyzed on the first 2-min of the Y-maze exposure. The variables used were the number of entries, the distance traveled and the time spent in each of the 3 arms. The entry in a given arm was considered when the rat went beyond the initial 10 cm of the arms.

### The Elevated-Plus-Maze

The Elevated-Plus-Maze (EPM) test evaluates mainly the anxiety level of rats[27]. It was carried out 2 days after the Y-maze in the same room. The EPM apparatus is a cross with 4 arms of 50 cm long and 10 cm wide, two arms are surrounded by a 40 cm high wall (closed arms) whereas the 2 others are deprived from wall (open arms). The device, transparent for infrared illumination, was placed above the infrared floor. The open arms were largely illuminated (450 Lux) but the closed arms were in a dim light (60 Lux). 

The rats were transferred from housing facilities to the testing room in their own cage. They were placed at the center of the cross, the nose oriented to the closed arms, and left for a 10-min free exploration. At the end, the number of defecations was counted and the apparatus carefully cleaned with soapy water and then a water/isopropyl alcohol (50%) solution. 

The behavior was recorded using the same videotracking system (Viewpoint, Lyon). The variables considered over the entire test duration were the following (i) the defecation bolus number, (ii) the number of entries in open and closed arms, (iii) the distance traveled in closed arms and (iv) the time spent in open arms.

### Sacrifice and tissue sampling

The rats were sacrificed in the morning after overnight fasting. They were transferred in their home cage to a dedicated room to avoid any additive stress. They were quickly anesthetized with a mixture of isofluran (3% in 100% O_2_). Blood samples were taken by cardiac puncture in a tube coated with lithium heparinate or EDTA. The brain was quickly removed and dissected on an ice bed using a glass tool. The entire hippocampus and the frontal cortex were sampled and immediately frozen in liquid nitrogen and stored at -80°C until analyses.

### mRNA determinations

Total RNA was isolated from hippocampus and cortex using Trizol reagent (Invitrogen, Carlsbad, CA). RNA concentrations and integrity were determined using RNA 6000 Nano Assay Kit and the Bioanalyzer 2100 according to the manufacturer’s instructions (Agilent Technologies, Santa Clara, CA). The primers used for PCR were as follows: *Ir* primers, 5’-CAAAAGCACAATCAGAGTGAGTATGAC-3’ and 5’-ACCACGTTGTGCAGGTAATCC-3’; *Irs1* primers, 5’GCCTGGAGTATTATGAGA ACGAGAA-3’ and 5’-GGGGATCGAGCGTTTGG-3’; *Irs2* primers, 5’-AAGATAGCGGGTACATGCGAAT -3’ and 5’- GCAGCTTAGGGTCTGGGTTCT -3’; *Glut1* primers, 5’-GTGCTTATGGGTTTCTCCAAA-3’ and 5’-GACACCTCCCCCACATACATG -3’; *Glut2* primers, 5’-TTTGCAGTAGGCGGAATGG-3’ and 5’- GCCAACATGGCTTTGATCCTT-3’; *Gys1* primers, 5’- TCCACTGTGCCTGTGTCTTCA-3’ and 5’-AGAGAACTTCTTCACATTCAGTCCATT-3’; *Gsk3b* primers, 5’- TTAAGGAAGGAAAAGGTGAATCGA-3’ and 5’- CCAAAAGCTGAAGGCTGCTG-3’; *18S* primers, 5’-TAAGTCCCTGCCCTTTGTACACA-3' and 5’-ATCCGAGGGCCTCACTA

AAC-3’. mRNA levels were assessed by real-time quantitative RT-PCR. All PCR reactions were performed in a total volume of 25 µl and included the following components: cDNA derived from 25 ng of total RNA, 400 nM of each primer, RNase-free water, and 12.5 µl of SYBR Green PCR Master Mix (ABI), an optimized buffer system containing AmpliTaq Gold DNA polymerase and dNTPs. All PCR reactions were performed in duplicate and cycling parameters were as follows: after an initial denaturation step for 10 min at 95°C, 40 subsequent cycles were performed in which samples were denatured for 15 s at 95°C followed by primer annealing and elongation at 60°C for 1 min. The relative quantities of mRNA were normalized by 18S rRNA content.

### Statistical analyses

Data are expressed as means ± SE. For the behavior testing, the statistical analyses were done using Statistica 7.1 software (Statsoft). The effect of conditioning was analyzed using a two-way factorial analysis with the Diet (High Fat/High Fructose vs. Control Diet) and the CN (Cinnamon vs. No-Cinnamon) effects and interactions. If necessary, post-hoc tests were carried out using the Bonferroni test for all couples. Differences in performance from one to another of Y-maze sessions were assessed using analysis of variance for repeated measurements. Differences in alternance rate were analyzed using Chi^2^ test. One-way analysis of variance (ANOVA) was used to determine the significance of the effects of diet and CN treatment on mRNA. When significant intergroup differences were found (P<0.05), the Tukey’s test was carried out. Different superscripts indicate significant differences among groups, P<0.05. 

## Results

In an associated study, we demonstrated that the animals fed the HF/HFr diet had decreased insulin sensitivity based upon the hyperinsulinemic euglycemic clamp procedure and that CN prevented the negative effects of the HF/HFr diet on insulin sensitivity [20]. 

### Y-maze

In this study, no difference in defecation number was observed among groups in either of the 2 sessions (Table 1). In the first session, CN supplementation was followed by an enhanced motor activity as suggested by the increase in distance traveled (P<0.05) and time spent in rearing . The same was observed in the second session (distance traveled, P<0.01 and rearing duration, P<0.01). A habituation to the device occurred in each group as the rats exhibited less defecation number, greater distance traveled and more time spent in rearing (session 1 *vs.* session 2, P<0.05 for each variable in each group).

**Table 1:** Behavioral performance in Y-maze.

		**C**	**C+CN**	**HF/HFr**	**HF/HFr+CN**
		**Mean SE**	**Mean SE**	**Mean SE**	**Mean SE**
**Defecation**	Session 1	1.7±0.5	1.2±0.4	1.9±0.6	2.5±0.7
	Session 2	0.7±0.3	0.6±0.3	0.7±0.3	1.1±0.3
**Distance**	Session 1	2209±151	2825±128	2467±144	2485±114 **^*#*^**
	Session 2	2417±174	3180±200	2501±254	2822±139 **^*##*^**
**Rearing duration**	Session 1	94±9	122±9	100±10	113±13
	Session 2	102±11	156±11	111±14	130±10 **^*##*^**

Behavioral data were obtained during the entire Y-maze test (sessions1 and 2) in Control (C), control plus cinnamon (C+CN), high fat/high fructose (HF/HFr) and high fat/high fructose diet plus cinnamon (HF/HFr+CN). There were 20 rats per group. Defecation, distance traveled (cm) and duration of rearing (sec) are expressed as mean±SE. Comparisons among groups were done using 2-way factorial ANOVA with cinnamon effect (***^#^***P<0.05 and ***^##^***P<0.01).

No differences among groups were observed in their propensity to choose the Unknown arm at the beginning of the second session of Y-maze: C:18 out of 20 rats, C+CN: 14 out of 20 rats, HF/HFr: 16 out of 20 rats and HF/HFr+CN: 15 out of 20 rats. Each group exhibited a performance far above random (at least P<0.05 for each group).

The activating effect of CN was also observed in the first 2-min period of the first session as CN rats had an increased distance traveled (Entry arm, P<0.05) (Table 2). This was also observed in the first 2-min period of the second session: CN rats exhibited an increased number of entries (Entry arm, P<0.05 and Known arm, P<0.05) and distance traveled (Known arm, P<0.01). The CN rats also presented an increased time spent in the Entry arm (P<0.05). No differences in interest for the Unknown arm were observed among groups: same number of entries, same time spent and distance traveled inside. Differences between the 2 sessions were observed differently according to groups. The C rats had reduced exploration of the Known arm (number of entries, P<0.001; time spent, P<0.05 and distance traveled, P<0.001). The C+CN rats had reduced exploration of both the Entry arm (number of entries, P<0.05; time spent, P<0.01 and distance traveled, P<0.001) and the Known arm (number of entries, P<0.01 and time spent, P<0.01). The HF/HFr rats limited their interactions with the Entry arm (number of entries, P<0.05 and distance traveled, P<0.001) and the Known arm (time spent, P<0.05 and distance traveled, P<0.01). The HF/HFr+CN rats only reduced their interaction with the Entry arm (time spent, P<0.01 and distance traveled, P<0.001).

**Table 2:** Behavioral performance in Y-maze.

		**C**	**C+CN**	**HF/HFr**	**HF/HFr+CN**
**Entry arm **		**Mean SE**	**Mean SE**	**Mean SE**	**Mean SE**
**Number of entries**	Session1	2.50±0.24	3.15±0.20	2.8±0.17	2.80±0.25
	Session 2	2.15±0.17	2.55±0.17**^*a*^**	2.15±0.15**^*a*^**	2.50±0.17**^*#*^**
**Time spent**	Session 1	54± 6	54±5	59± 5	67± 6
	Session 2	50± 7	27± 3**^*b*^**	47±7	40±6**^*#*^** ^ b^
**Distance traveled**	Session 1	190±18	247±13	227±14	234±13**^*#*^**
	Session 2	155±13	164±16**^*c*^**	158±9 **^*c*^**	159±11**^*c*^**
**Known arm**					
**Number of entries**	Session 1	1.75±0.22	2.40±0.23	1.85± 0.2	2.00±0.25
	Session 2	1.00± 0.13 **^*c*^**	1.75± 0.19 **^*b*^**	1.5±0.28	1.70±0.23 **^*#*^**
**Time spent**	Session 1	40±6	35±3	30±4	31±5
	Session 2	22±7 **^*a*^**	28±5 **^*b*^**	18±3 **^*a*^**	21±3
**Distance traveled**	Session 1	157±14	193±17	149±13	143±16
	Session 2	81±11 **^*c*^**	142±13	100±17 **^*b*^**	117±15**^*##*^**
**Unknown arm**					
**Number of entries**	Session 2	2.45±0.34	3.00±0.28	2.2±0.24	2.50±0.29
**Time spent**	Session 2	24± 3	30±4	25±3	24±3
**Distance traveled**	Session 2	164±21	202±15	160±18	169±19

Behavioral data were obtained during the first 120 sec of Y-maze test (session 1 and 2) in Control (C), control plus cinnamon (C+CN), high fat/high fructose (HF/HFr) and high fat/high fructose diet plus cinnamon (HF/HFr+CN) groups. There were 20 rats per group. Number of entries, the time spent (sec) and distance traveled (cm) in each of the 3 arms are expressed as mean±SE. Comparisons among groups were done using 2-way factorial ANOVA with cinnamon effect (***^#^***P<0.05 and ***^##^***P<0.01). Comparison between sessions were done in each group using ANOVA for repeated measurements (***^a^***P<0.05; ***^b^***P<0.01; ***^c^***P<0.001)

### Elevated-plus-maze

The rats having CN supplementation had higher defecation number than the rats fed without CN (p<0.05) (Table 3). The rats fed with HF/HFr entered less (p<0.05), spent less time (p<0.05) and carried out less locomotion (p<0.05) in the open arms than the rats fed with C diet. No differences of locomotion, number of entries and time spent were observed among groups in the closed arms.

**Table 3:** Behavioral performance in Elevated Plus Maze test.

**Diet**	**C**	**C+CN**	**HF/HFr**	**HF/HFr+CN**
	**Mean SE**	**Mean SE**	**Mean SE**	**Mean SE**
**Defecations**	0.6±0.3	2.1±0.6	0.8±0.4	1.6±0.4 **^*#*^**
**Open arms**				
**Entries**	6.9±1.3	6.9±0.9	4.4±0.7 *	5.3±0.7
**Time spent**	70±17	67± 11	36±8 *	43±8
**Distance**	416±108	410±63	211±50 *	256± 42
**Closed arms**				
**Entries**	17.8±1.5	17.7±0.9	13.9±1.7	18.7±1.7
**Time spent**	465±23	480±15	515±14	493±12
**Distance**	1811±74	1860±87	1782±134	1948±114

Behavioral data obtained during Elevated-Plus Maze test in Control (C), control plus cinnamon (C+CN), high fat/high fructose diet (HF/HFr) and high fat/high fructose diet plus cinnamon (HF/HFr+CN) groups. Number of defecations in the entire device, the number of entries, and the time spent (sec) and distance traveled (cm) in the open and closed arms are expressed as mean ± SE. Comparisons were done using 2-way factorial ANOVA with diet effect (^*^P<0.05) and cinnamon effect (***^#^*** P<0.05).

### mRNA effects

**Figure 1:**
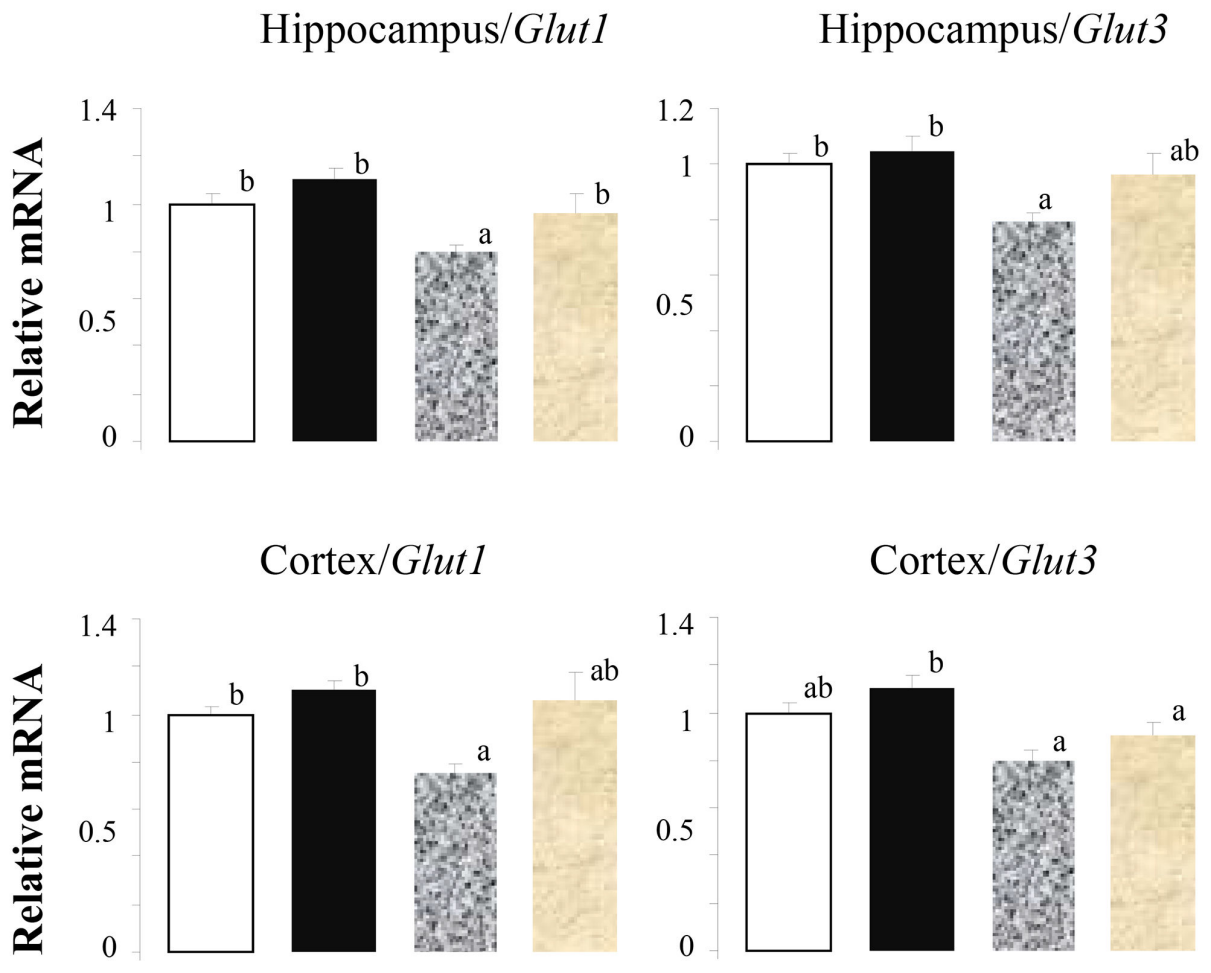
High Fat/High Fructose (HF/HFr) diet decreases *Glut 1* and 3 in hippocampus and cortex. Open bar, control diet; solid bar, control diet plus cinnamon, mottled gray bar, HF/HFr diet; gray bar, HF/HFr diet plus cinnamon. Values are mean ± SE for 8 to 10 rats. Different letters denote significant differences among groups, p<0.05.

Consistent with the negative effects of the HF/HFr diet on insulin sensitivity, and behavior, there were also decreases in mRNA for glucose transporters in the hippocampus and cortex. Animals consuming the HF/HFr diet had a decrease in mRNA coding for GLUT1, the principal glucose transporter of the blood-brain barrier, in both the hippocampus and cortex that was prevented by the CN (Figure 1, left panel). mRNA coding for GLUT3, the main facilitative glucose transporter in neurons, was also decreased by the HF/HFr diet in the cortex with a return to the level of the control diet in the animals consuming the control diet plus CN (Figure 1, right panel).

**Figure 2:**
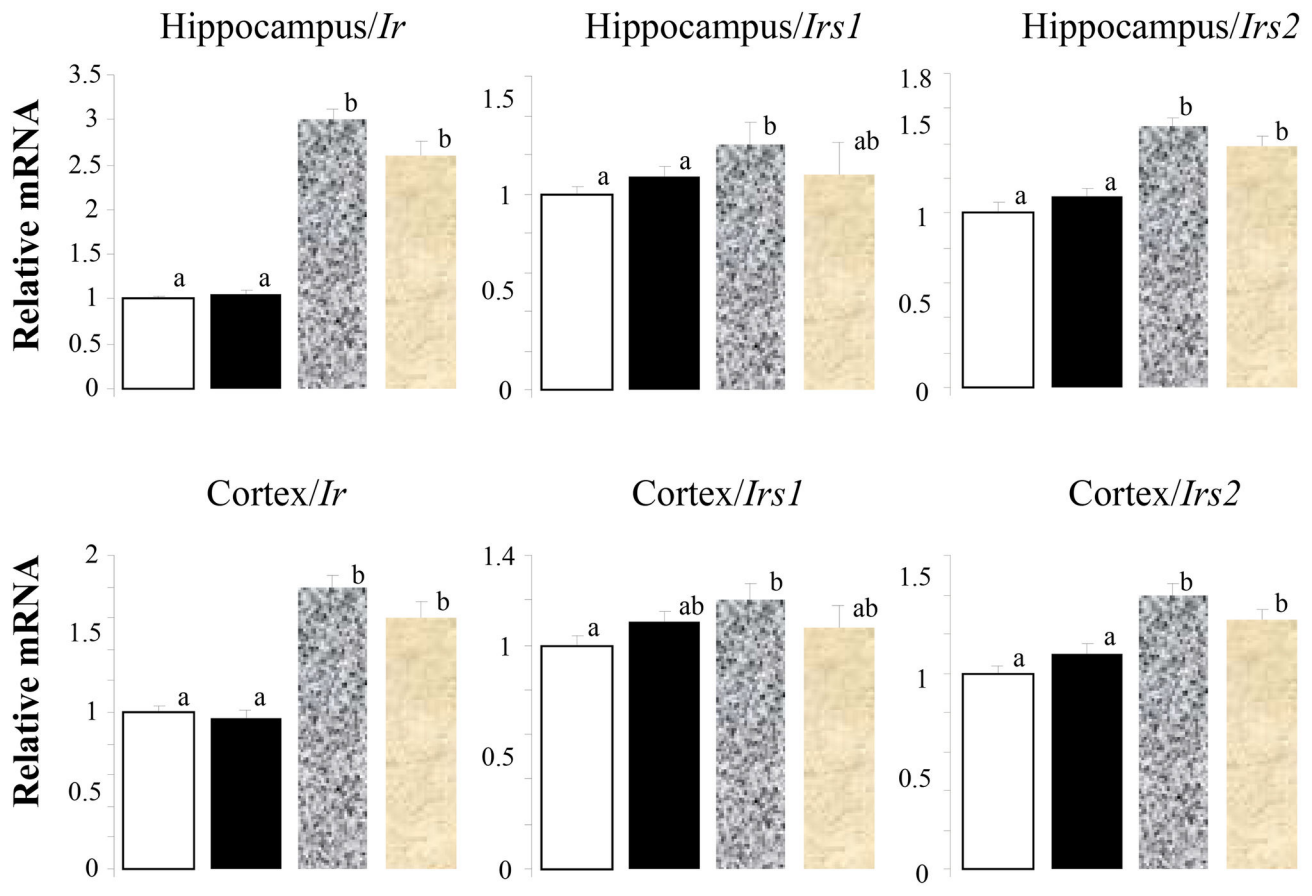
High Fat/High Fructose diet increases *Insr*, *Irs1* and *Irs2* in hippocampus and cortex. Open bar, control diet; solid bar, control diet plus cinnamon, mottled gray bar, HF/HFr diet; gray bar, HF/HFr diet plus cinnamon. Values are mean ± SE for 8 to 10 rats. Different letters denote significant differences among groups, p<0.05.

Contrary to that observed in the muscle [21], consumption of the HF/HFr diet led to increases in insulin receptor (Ir), *Irs1* and *2* in the hippocampus and cortex that were not reversed by CN (Figure 2) demonstrating that changes in these variables in the hippocampus and cortex were not associated with similar changes in the periphery. Associated negative effects on these variables related to insulin resistance requires further study. 

**Figure 3:**
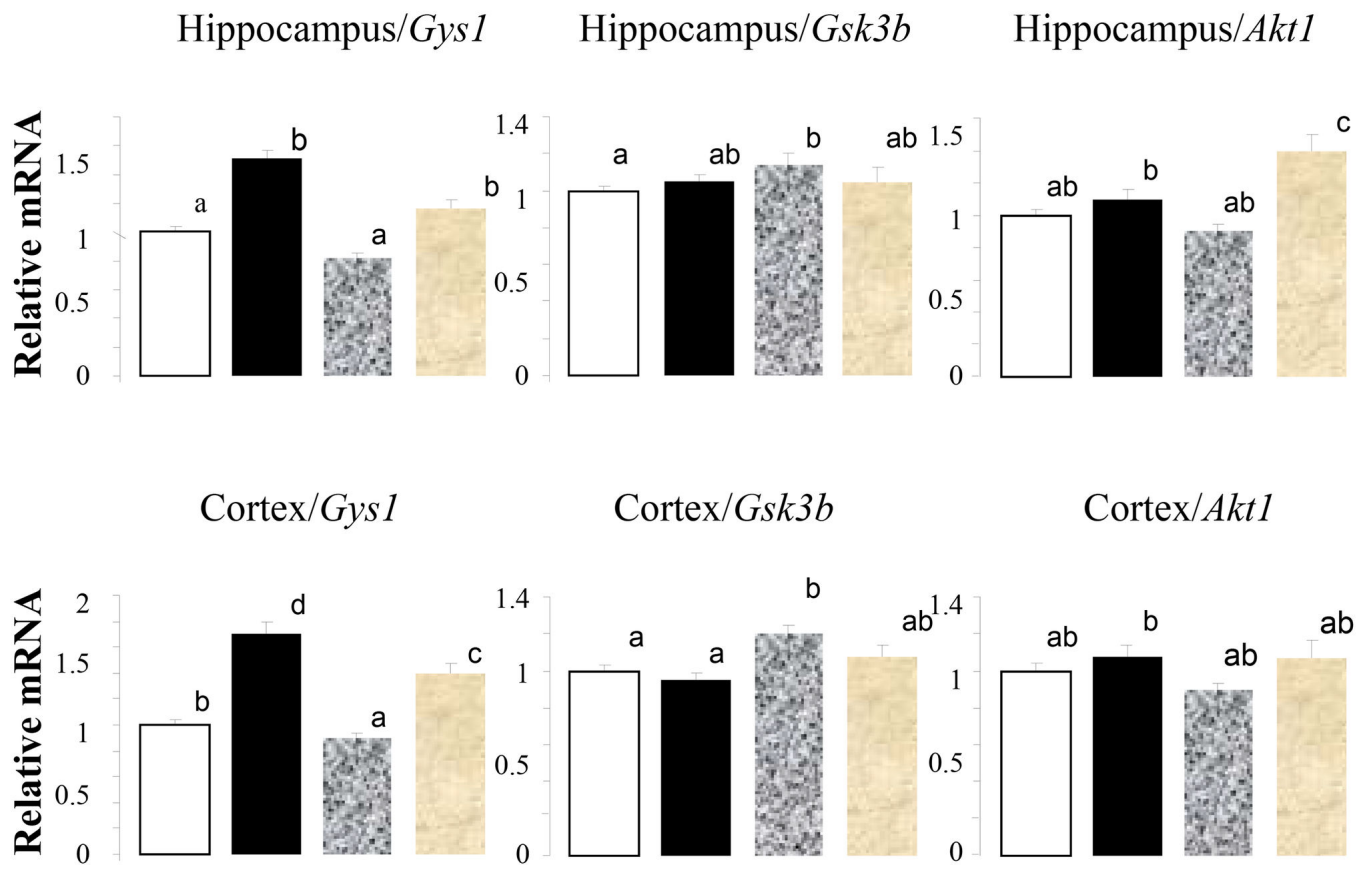
High Fat/High Fructose diet effects on *Gys1*, *Gsk3β* and *Akt1* in hippocampus and cortex. Open bar, control diet; solid bar, control diet plus cinnamon, mottled gray bar, HF/HFr diet; gray bar, HF/HFr diet plus cinnamon. Values are mean ± SE for 8 to 10 rats. Different letters denote significant differences among groups, p<0.05.

There were significant effects due to CN on the mRNA coding for glycogen synthase, *Gys1*, in animals consuming both the control diet as well as those consuming the HF/HFr diet in both the hippocampus and cortex (Figure 3, left panel). Changes in the mRNA coding for glycogen synthase kinase (GSK-3β), which is involved in the control of the activity of glycogen synthase, were increased in animals consuming the HF/HFr diet and not altered by CN in animals consuming either the control or HF/HFr diet in either the hippocampus or cortex (Figure 3, middle panel). Increases in mRNA coding for protein kinase B (AKT1), a key control step in insulin signaling, were significant in animals consuming the HF/HFr diet plus CN in the hippocampus but not the cortex (Figure 3, right panel).

**Figure 4:**
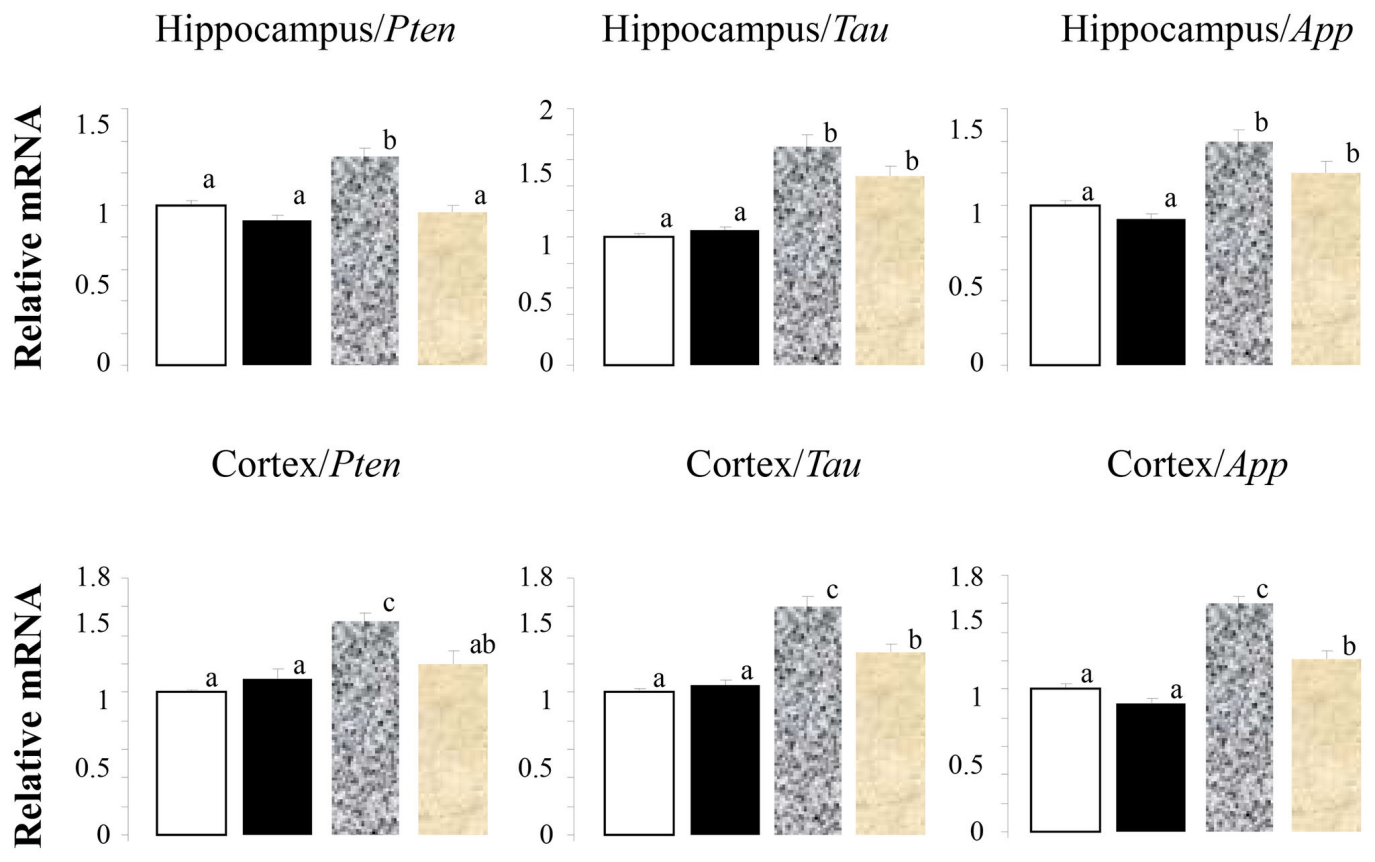
High Fat/High Fructose diet increases *Pten*, *Tau* and *App* in hippocampus and cortex. Open bar, control diet; solid bar, control diet plus cinnamon, mottled gray bar, HF/HFr diet; gray bar, HF/HFr diet plus cinnamon. Values are mean ± SE for 8 to 10 rats. Different letters denote significant differences among groups, p<0.05.

The negative effects of the HF/HFr diet on insulin sensitivity were associated with negative effects on mRNA coding for proteins associated with Alzheimer’s disease and memory loss including phosphatase and tensin homolog (*Pten*), *Tau* and amyloid precursor protein (App) (Figure 4). The HF/HFr diet led to increases in *Pten* (Figure 4, left panel), *Tau* (Figure 4, middle panel) and *App* (Figure 4, right panel) that were reversed by CN. Increases in these variables are associated with decreased insulin sensitivity . Cinnamon had no detectable significant effects on these variables in animals consuming the control diet. There were no detectable effects of CN on the animals consuming the control diet plus CN. 

## Discussion

Cinnamon is often used to refer to Ceylon cinnamon or “true cinnamon” (*Cinnamomum verum*) however, related species of cassia (*C. aromaticum and C. burmannii*) are often sold as CN. Cinnamon was first imported to Egypt in 2000 BC and it is often referred to in the bible [19]. While the taste and coumarin content often differ substantially among cinnamons, many of the properties and effects of the true CN and related species are similar. Cinnamon has a long history of uses as a spice, flavoring agent, preservative, and pharmacological agent. More recent studies have documented the role of CN in the prevention of insulin resistance, metabolic syndrome and type 2 diabetes (see review) [19]. In addition, CN has been shown to alleviate factors associated with Alzheimer’s disease and memory loss by blocking and reversing TAU formation and blocking the effects of amyloid precursor protein [28]. The common link between Alzheimer-like changes and type 2 diabetes may be insulin resistance. The peripheral insulin-sensitizer drug, metformin, ameliorates neuronal insulin resistance and Alzheimer-like changes [5]. Evidence that insulin signaling mechanisms are important for neuronal survival [9,29] and studies demonstrating reduced expression of the insulin receptor in the brain of patients suffering from AD have generated increased interest in determining the role of impaired insulin actions associated with Alzheimer-like changes [5]. The fact that insulin receptors are widely expressed in the brain, combined with the observation that insulin crosses the blood-brain barrier by a saturable, receptor mediated transport mechanism, further highlights the observations that insulin likely plays a key role in memory and cognition [30,31]. Defective insulin signaling is associated with decreased cognitive ability and development of dementia. Neurons are insulin-dependent metabolically active tissues, like fat and muscle, that develop insulin resistance and thus cannot respond properly to the neurotrophic properties of insulin, resulting in neuronal injury, subsequent dysfunction and ultimately Alzheimer’s and related diseases [32].

Impaired insulin sensitivity, measured by HOMA, is associated with deficits in verbal fluency [33] and it is not necessary to wait to have overt signs of AD to detect changes in behavior, memory and cognition. Short-term memory alteration has been found in the early phases of AD [34] and in diets high in fat [35]. In addition, HbA1c is associated with executive functioning and working memory in people with insulin resistance [36] and CN also improves HbA1c [37-39]. 

In this study, we measured the effects of early changes in insulin sensitivity and the counteracting insulin sensitizing effects of CN, on changes in behavior and mRNA associated with insulin signaling and Alzheimer’s. The HF/HFr diet is known to induce changes in behavior [40,41], memory and cognition [42,43]. In our study, the HF/HFr did not induce changes in short-term memory as detected using the Y-maze. Animals fed with HF/HFr diet exhibited the same probability as rats fed with the control chow diet of spontaneous choice of unexplored arm in the second session of Y-maze. Further, they showed the same level of habituation to testing conditions as suggested by the decrease in defecation number and the enhanced locomotion in the second session compared to the first one. 

In the elevated plus maze, the HF/HFr fed rats exhibited decreased exploration of the open arm. The reduced exploration of open arms cannot be explained by a decrease in locomotion since the explorations of closed arms were not different among groups. It would be better explained by an enhanced anxiety level [44]. The effect of HF/HFr on anxiety is congruent to the anxiogenic effect of a diet rich in sucrose [45] and with increased stress reactivity induced by a HF diet [46]. However, the latter effect is not always observed, especially in case of a short term HF diet [47]. The anxiety level of HF/HFr rats seems context-dependent as it was observed in EPM but not in Y-maze when HF/HFr rats coped with the Unknown arm during the second session. The differences between both behaviors may be related to the level of aggressiveness as Y-maze Unknown arm is more secure than the open arm of EPM due to the wall and the dim light.

The HF/HFr diet used in this study has been shown to induce an impairment of insulin-sensitivity in muscle and liver [21]. We also observed brain mRNA expression alterations indicative of insulin resistance in this study (Figures 1-4). Such alterations might have behavioral counterparts. Since insulin crosses the blood-brain barrier by a saturable, receptor mediated transport mechanism and acts on receptors widely expressed in the brain [48], it may modulate brain functions such as memory and cognition [30,31,49]. Impaired insulin sensitivity would be one relevant hypothesis to explain HF/HFr diet-induced behavioral alterations. In humans, impaired insulin sensitivity, as measured by HOMA score, is associated with deficits in verbal fluency [33] and intranasal insulin increases verbal memory in humans [50]. HbA1c is associated with executive functioning and working memory in people with insulin resistance [36]. In animals, impaired insulin-sensitivity is also associated with behavioral perturbations [40,42,45] while intracerebroventricular insulin increases memory in rodents [51]. The hippocampus is one of the potential targeted brain areas, as insulin receptors are found at high densities [31]. Furthermore, the hippocampus inhibits stress reaction and supports explicit and spatial memory [33]; all functions being modulated by insulin effects. One hypothesis would be that insulin resistance in hippocampus might explain the enhanced anxiety. Alternatively, the frontal cortex would be also questioned as insulin receptors are also highly expressed [48] and working memory is impaired in case of insulin resistance [36]. This is in agreement with the fact that insulin sensitivity is also impaired in frontal cortex area. 

The mechanisms by which insulin sensitivity may act on brain deserve discussion. Brain is an insulin-dependent metabolically active tissue, like fat and muscle. Insulin may therefore act through its metabolic effects. The exposure to a challenging environment is associated with brain activation during which an increase in breakdown of glycogen occurs [52]. The rapid increase in glycogen turnover appears important for memory formation as supported by the memory loss after inhibition of glycogen turnover [53]. Insulin, which favors glycogen turnover [54] and improves memory [48,51], may therefore act on brain function through its metabolic effects. The same can be concluded for frontal cortex as insulin increases working memory [55]. Decreases in insulin sensitivity were associated with the anticipated decreases in Glut 1, in both the hippocampus and cortex. GLUT1 is highly expressed in endothelial cells of blood brain barrier and is responsible for transporting glucose from the blood to extracellular space of the brain [56]. Alternatively, insulin may act through its effect on neurotrophicity [7,48]. There is evidence that insulin signaling mechanisms are important for neuronal survival [9,29]. In the case of insulin resistance, the brain cannot respond properly to the neurotrophic properties of insulin, resulting in neuronal injury, subsequent dysfunction and ultimately Alzheimer’s and related diseases [32].

We observed several mRNA expression changes supporting the hypothesis that insulin resistance might be associated with the occurrence of AD brain markers. The increase in TAU filaments is associated with extensive deposition of amyloid β and a strong association with AD. The amyloid β hypothesis states that amyloid β deposition directly affects neurons, including neurofibrillary tangles and neuronal death, leading to memory impairment which is one of the key clinical signs of AD-related brain dysfunction. Mice engineered to over express mutant APP exhibit increased APP deposition and memory impairment [57]. However, reducing amyloid β generation or removing deposits failed to halt the progression of dementia [58,59]. Consistent with these studies, we observed an increase in mRNA coding for TAU and APP in both hippocampus and frontal cortex. GSK-3β intervenes before neurofibrillary tangles are formed since GSK-3β phosphorylates TAU protein, a step leading to granular TAU oligomers, then neuronal dysfunction and synaptic loss [58]. 

Recent studies have documented the role of CN in the prevention of insulin resistance, metabolic syndrome and type 2 diabetes (see review) [19]. CN also inhibits the misfolding of human islet amyloid polypeptide which is regarded as a causative factor for type 2 diabetes mellitus [60]. CN supplementation reverses some, but not all, the deleterious effects of the HF/HFr diet in liver and muscle [21]. In muscle, there were no significant effects of CN on *Insr, Irs1* and *Irs2* in animals consuming the control diet whereas all three were reduced in the animals consuming the HF/HFr diet. Negative effects of the HF/HFr diet were prevented in the animals consuming the HF/HFr diet plus CN. Contrary to the effects observed for muscle, in the hippocampus and cortex, consumption of the HF/HFr diet led to increases in *Ir, Irs1* and *Irs2* that were not prevented by the consumption of CN (Figure 2). This apparent anomaly is consistent with the study demonstrating that IRS 2 is a negative regulator of memory function and restricts dendritic spine generation [61]. Deletion of *Irs2* reduces amyloid deposition and rescues behavioral deficits in APP transgenic mice [62] and increases in *Irs2* in this study are consistent with negative effects on learning and memory. Although in a nonsignificative manner, cinnamon blunted the HF/HFr diet effects on anxiety in EPM. 

Cinnamon also prevented the decreases in *Glut1* in animals consuming the HF/HFr diet plus CN. Similar effects were observed for *Glut3*, also named the “brain glucose transporter” because it is expressed at high levels in nerves and neural tissue [63]. Cinnamon increased the expression of *Gys1* not only in the control animals but also reversed the decreases in *Gys1* expression in the cortex in the animals consuming the HF/HFr diet. This suggests that cinnamon may have corrected some metabolic effects of insulin resistance. 

In addition, CN alleviated the negative effects of the HF/HFr diet on Alzheimer-related variables in the brain that we analyzed. Recent studies showed that CN alleviates factors associated with Alzheimer’s disease and memory loss by blocking and reversing TAU formation and blocking the effects of amyloid precursor protein [28]. An aqueous extract of Ceylon cinnamon (*C. zeylanicum*) was shown to inhibit hallmark signs of AD including TAU aggregation and the formation of TAU filaments [16]. TAU filaments removed from the brain of a person who died with AD were also disassociated by a CN extract. A type A-linked procyanidin trimer purified from the aqueous extract was shown to contain a significant portion of the inhibitory activity. This type A trimer has subsequently been purified and identified as cinnamtannin B-1, MW 864 (Richard Anderson, unpublished results). However, there are other active components in cinnamon and Lu et al. [64] demonstrated that both type A- and B-type proanthocyanidins may improve insulin sensitivity. Cinnamon proanthocyanidins were also shown to be the major anti-diabetic components of a cinnamon water extract in the prevention of the misfolding of human islet amyloid polypeptide, a proposed causative factor for type 2 diabetes [60].

A limitation of this study is that additional measurements involving protein levels and protein modification levels were not measured. This study was designed to demonstrate that changes in insulin sensitivity due to diet and cinnamon are associated with changes in behavior and changes in the brain related to insulin sensitivity. This objective was accomplished, however, future studies should include protein related changes.

## Conclusion

In summary, this study demonstrated that short-term changes in diet led to not only changes in insulin sensitivity and behavior but also early changes in mRNA coding for proteins related to memory including glycogen synthase kinase 3β, glycogen synthase, PTEN, TAU, and APP that could be prevented or alleviated due to the intake of CN. The CN fed rats were more active in a Y maze test than rats fed the control and HF/HFr diets. The HF/HFr diet fed rats showed greater anxiety in an elevated plus maze test that was lessened by feeding CN. In conclusion, the negative effects of a HF/HFr diet on brain insulin signaling and behavior were alleviated by CN suggesting neuroprotective effects of CN associated with whole body improved insulin sensitivity and related changes in the brain. The consumption of foods or diets that lead to decreased insulin sensitivity may lead not only to changes in insulin related variables but also possible early changes in behavior that can be prevented or alleviated by CN. Additional studies are needed to further characterize the proteins and metabolites associated with these changes. 
